# Nanocarrier-Loaded Imidaclothiz Promotes Plant Uptake and Decreases Pesticide Residue

**DOI:** 10.3390/ijms23126651

**Published:** 2022-06-14

**Authors:** Qinhong Jiang, Min Peng, Meizhen Yin, Jie Shen, Shuo Yan

**Affiliations:** 1 Department of Plant Biosecurity and MARA Key Laboratory of Surveillance and Management for Plant Quarantine Pests, College of Plant Protection, China Agricultural University, Beijing 100193, China; j.yucheng@outlook.com; 2 State Key Laboratory of Chemical Resource Engineering, Beijing Laboratory of Biomedical Materials, Beijing University of Chemical Technology, Beijing 100029, China; 2020400119@buct.edu.cn (M.P.); yinmz@mail.buct.edu.cn (M.Y.)

**Keywords:** nano-delivery system, nanopesticide, pesticide adjuvant, polymer, sustainable agriculture

## Abstract

There is a great demand for improving the effective utilization of pesticides and reducing their application for sustainable agriculture, and polymeric nanoparticles have provided strong technical support for the efficient delivery of pesticides. To this context, we tried to construct a relatively safe imidaclothiz nano-delivery system for enhanced plant uptake, reduced pesticide residue and improved bioactivity toward green peach aphids. The imidaclothiz could be assembled with the hydrophobic core of SPc through hydrophobic association, which led to the self-assembly of nanoscale imidaclothiz/SPc complex consisting of nearly spherical particles. The SPc decreased the contact angle of imidaclothiz drops and remarkably increased the plant uptake. Furthermore, the bioactivity and control efficacy of imidaclothiz were significantly improved with the help of SPc in both laboratory and field. Excitingly, the residue of imidaclothiz decreased with the help of SPc 7 d after the treatment due to the faster degradation of nanoscale imidaclothiz/SPc complex, which exhibited no negative effects on agronomic traits of tobacco plants. The current study successfully constructed a nano-delivery system for imidaclothiz, which can not only increase the effective utilization of pesticides, but also decrease the pesticide residue.

## 1. Introduction

In recent years, nanotechnology has provided strong technical supports and innovative ideas for sustainable agriculture, and a series of nanoparticles have been designed and constructed as carriers of synthetic/botanical pesticides and fertilizers [1,2,3,4,5]. Most synthetic pesticides contain the hydrophobic active ingredients (AIs) that can be encapsulated in or attached to the peripheral groups of nanoparticles [6,7,8]. Polymeric nanomaterials have been recently applied for agrochemical delivery, and it is important to explore efficient polymer nanocarriers. A star polymer (SPc) has been designed and synthesized to deliver both double-stranded RNA (dsRNA) and synthetic/botanical pesticides [9,10,11,12,13]. The SPc can activate the clathrin-mediated endocytosis to improve the delivery efficiency of loaded cargo [14,15]. Additionally, the plant-uptake of SPc-loaded pesticides is significantly improved, while the pesticide residue is simultaneously decreased [16,17,18]. In a recent publication, the SPc has been successfully applied to co-deliver dsRNA and botanical pesticide to overcome the short life disadvantage of dsRNA and slow-acting property of botanical pesticide for a great enhancement of sequential bioactivity [19]. Therefore, SPc exhibits good potential for field application as an excellent adjuvant for improving the delivery efficiency of various agents for plant protection.

As a highly effective pesticide, neonicotinoid pesticide has become one of the most heavily applied classes of insecticides worldwide since the 1990s, with the large-scale application including plant protection (crops, vegetables and fruits), veterinary products and biocides to invertebrate pest control in fish farming [20]. Neonicotinoids act as a competitive inhibitor on nicotinic acetylcholine receptors in the central nervous system, and their systemic properties and long residual activity make them ideal insecticides toward sucking pests [21]. However, their residual levels are relatively high in the environment, and the excessive application of neonicotinoids has led to food safety problems [22,23,24,25]. Furthermore, the neonicotinoids exhibit toxicity toward non-target organisms such as bees, earthworms, predatory lady beetles, etc. [26,27,28]. As a fourth-generation neonicotinoid insecticide, the imidaclothiz, 1-(2-chloros-5-thiazolylmethyl)-N-nitroimida-zolin-2-ylideneamine is developed by Nantong Jiangshan Agrochemical Co., Ltd., and registered for the management of sucking and biting insects such as aphids, whiteflies, beetles and some Lepidoptera species [29]. The nano approach is a good choice to further increase the effective utilization of imidaclothiz. However, no information of imidaclothiz is available in this area.

The application of SPc has the potential to achieve the nanometerization of imidaclothiz and overcome its delivery obstacle for enhanced bioactivity. However, new environmental and human health hazards may emerge from SPc application, and several important issues should be firstly evaluated, including the pesticide residue, surface runoff and spray drift, hazard toward non-target predators, potential chemical damage toward plants, etc. [30,31,32,33,34]. To this context, we tried to construct a relatively safe imidaclothiz nano-delivery system for enhanced plant uptake and bioactivity toward aphids. The self-assembly mechanism of imidaclothiz/SPc complex was elucidated by determining the pesticide loading content (PLC) of SPc, particle size and morphology of imidaclothiz/SPc complex, and interaction between SPc and imidaclothiz. Then, the mechanism of enhanced bioactivity of imidaclothiz/SPc complex was illustrated by determining the contact angle, plant uptake and toxicity of dinotefuran/SPc complex. Finally, the environmental safety of imidaclothiz/SPc complex was demonstrated by testing the residue, toxicity toward non-target predatory lady beetles, and potential chemical damage toward tobacco plants.

## 2. Result and Discussion

### 2.1. Self-Assembly of Imidaclothiz/SPc Complex through Hydrophobic Interaction

**Figure 1:**
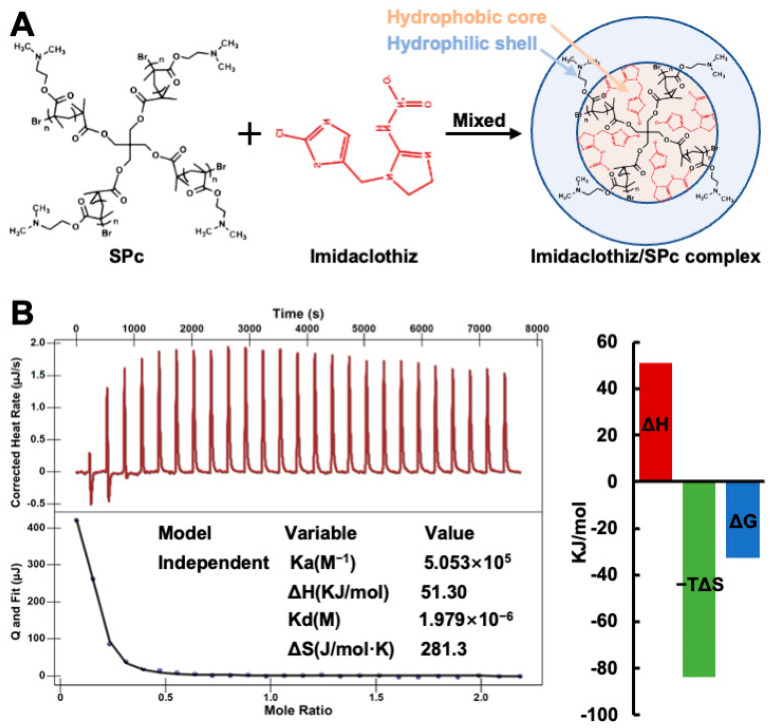
Schematic illustration of imidaclothiz/SPc complex (**A**) and ITC titration of SPc into imidaclothiz solution (**B**). The 2 mL of pure imidaclothiz solution (0.138 mmol/L) was titrated with 250 μL of SPc solution (1 mmol/L), and the test temperature was 25 °C.

The SPc is consisted of a hydrophilic shell with positively charged tertiary amines and a hydrophobic core, and the particle size of SPc is 100.5 nm [9]. The hydrophobic core is designed to combine with hydrophobic AIs, and the hydrophilic shell is beneficial for improving the water solubility and dispersion stability of loaded AIs [10]. As expected, the self-assembly of imidaclothiz/SPc complex could be easily realized through a simple mix and incubation at room temperature for 15 min. The imidaclothiz concentration was proportional to the ultraviolet absorption at 270 nm, and the PLC was calculated to be 16.31% (Figure S1), which was a bit lower than those of dinotefuran (17.41%), osthole (17.09%) and thiamethoxam (20.63%) [16,17,18]. The loading efficiency of SPc is comparable to those of other polymer-based nanocarriers [35,36]. The interaction of imidaclothiz with SPc was also analyzed using the isothermal titration calorimetry (ITC) according to the previous study (Figure 1) [37]. The negative ΔG revealed that the self-assembly was automatic, and high affinity constant K_a_ of 5.053 × 10^5^ M^−1^ and low dissociation constant K_d_ of 1.979 × 10^−6^ M suggested that this interaction was strong. The positive values of ΔH and ΔS demonstrated that the complexation of SPc with imidaclothiz was through hydrophobic association, revealing that the imidaclothiz was assembled in the hydrophobic core of SPc. Based on the current and our previous studies, the SPc can assemble with exogenous agents through different interactions [16,17,18], which is beneficial for expanding the application area of SPc.

### 2.2. Reduced Particle Size and Characterization of Imidaclothiz/SPc Complex

**Figure 2:**
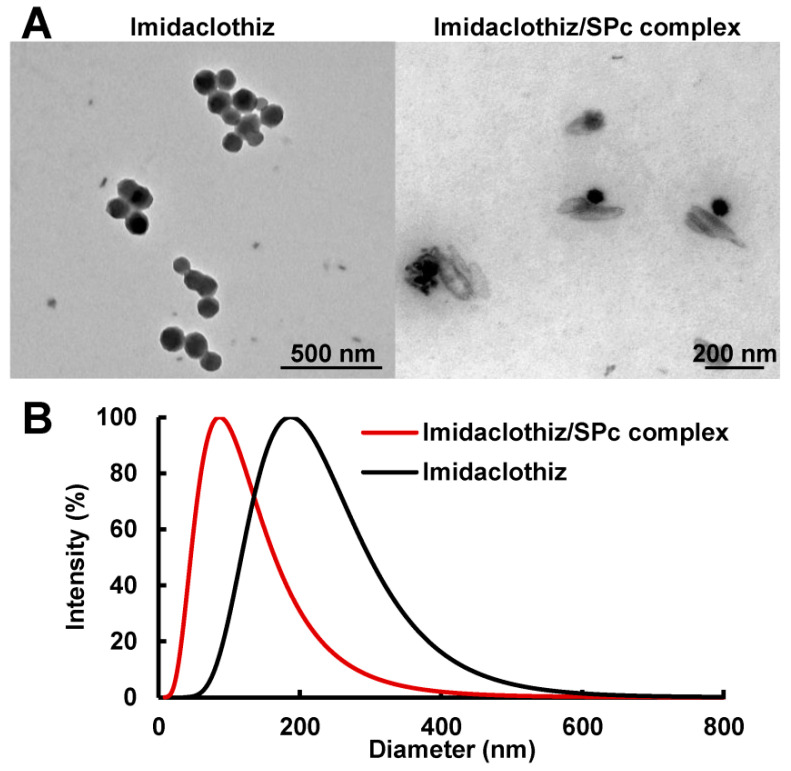
TEM images (**A**) and particle size distributions (**B**) of imidaclothiz and imidaclothiz/SPc complex at the mass ratio of 1:1.

As shown in Figure 2B and Table 1, the complexation of imidaclothiz with SPc disturbed the self-aggregated structure of imidaclothiz in aqueous solution, decreasing the particle size of imidaclothiz from 187.76 to 84.28 nm (mass ratio of 1:1). The particle size of imidaclothiz/SPc complex could be further reduced to 44.84 nm with the decreasing mass ratio. This conclusion was also supported by the representative transmission electron microscope (TEM) images (Figure 2A). It could be concluded that the most of self-aggregated imidaclothiz/SPc complex was composed of stable spherical particles with smaller size compared to imidaclothiz alone. The SPc can be applied as a universal adjuvant for pesticide nanometerization, and it is the first attempt to construct an imidaclothiz nano-delivery system to our knowledge [16,17,18]. The smaller particle size of polymer-loaded pesticide is beneficial for not only improving the systematic transmission and plant uptake of pesticide, but also increasing the contact area of pesticide to target pests for enhanced bioactivity [38,39,40].

### 2.3. Reduced Contact Angle and Increased Plant Uptake of Imidaclothiz/SPc Complex

**Figure 3:**
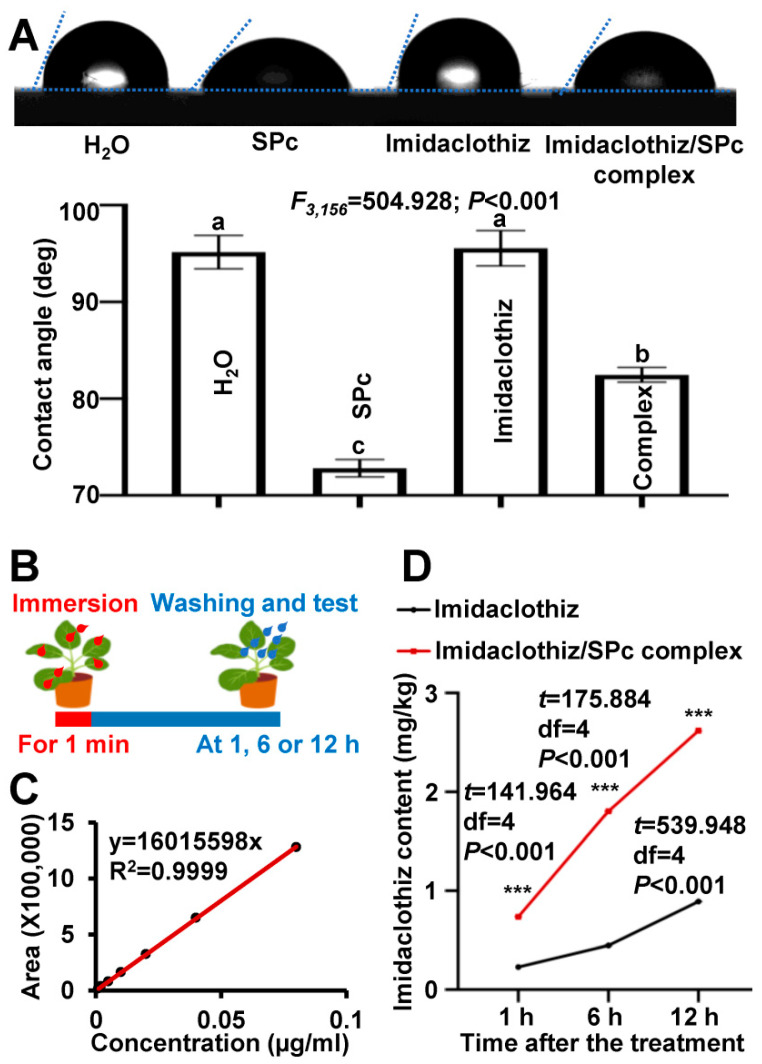
Contact angles (**A**) and plant uptake (**B**–**D**) of imidaclothiz and imidaclothiz/SPc complex at the mass ratio of 1:1. (**A**) Photos and correlation analysis of contact angles of various formulations. Each treatment contained 3 independent samples. Different letters indicate significant differences (Tukey HSD test, *p* < 0.05). (**B**) Schematic diagram for plant uptake assay. (**C**) Standard calibration curve of imidaclothiz for liquid chromatography-tandem mass spectrometry. (**D**) Imidaclothiz content in plants treated with imidaclothiz or imidaclothiz/SPc complex. Each treatment contained 3 independent samples. The “***” indicates significant differences (independent *t* test, *p* < 0.001).

The hydrophobic surface of plant leaves results in pesticide drift and environmental pollution [41,42]. After 10 s of contact, the contact angle of SPc-loaded imidaclothiz decreased from 95.56° to 82.48° (Figure 3A). Normally grown leaves carry a net negative charge, and the SPc with positively charged tertiary amines was more likely to be wetted on plant leaves. Furthermore, the SPc could reduce the surface tension of the imidaclothiz/SPc complex droplet to promote its spread and adhesion. Nanoparticles have been designed and applied as pesticide carriers for reduced contact angle and surface tension, and enhanced retention [43,44]. For instance, Chen et al. [45] has modified zein with dialdehyde carboxymethyl cellulose (DCMC) to construct a pesticide delivery system which can decrease the contact angle of loaded avermectin (AVM) and regulate the contact angle by adjusting the mass ratio of zein to DCMC.

Nano-delivery system has been applied to promote the transportation of pesticides in plants. The AVM can be detected in stems and all leaves of rice plants treated with nanocarrier-loaded AVM, whereas rare AVM was detected only in treated leaves for AVM alone, revealing the enhanced transportation [46]. Similarly to the same class of imidacloprid, due to its high water solubility and good transportation in plants, imidaclothiz can be taken up by plant roots and translocated upward, leading to relative enrichment in leaves [47]. In the current study, the imidaclothiz contents in tobacco plants treated with imidaclothiz were 0.23, 0.45 and 0.89 mg/kg at 1, 6 and 12 h after the imidaclothiz immersion, and those increased to 0.74, 1.80 and 2.62 mg/kg with the help of SPc (Figure 3B and Figure S2). The plant uptake of SPc-loaded imidaclothiz was remarkably improved 2.94–4.00 times, which might be related to the smaller particle size and contact angle of imidaclothiz/SPc complex. The mechanism of SPc-based enhanced cellular uptake has been elucidated in our previous studies. The SPc-loaded chitosan can activate the endocytosis pathway of potato plants by up-regulating *CHMP5*, *Epsin*, *Rab7* gene, etc. [14]. The clathrin-mediated endocytosis is the major route for SPc-mediated exogenous substance delivery, and the SPc can remarkably improve the delivery efficiency of loaded cargo [15].

### 2.4. Improved Bioactivity of SPc-Loaded Imidaclothiz toward Green Peach Aphids

**Figure 4:**
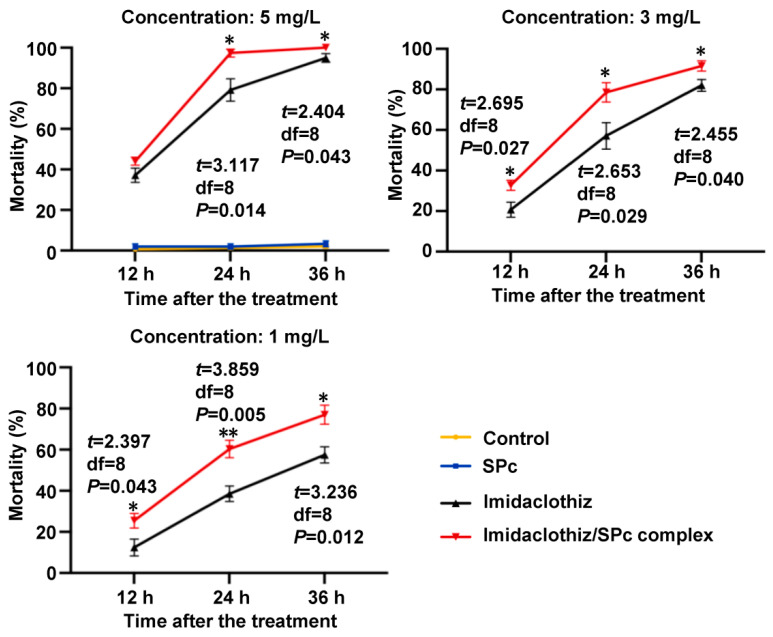
Bioactivity of imidaclothiz/SPc complex toward green peach aphids through root application. The pure imidaclothiz was mixed with SPc at the mass ratio of 1:5.1 according to the PLC. The highest concentrations of SPc and ddH_2_O were used as controls. Each treatment included approximately 30 aphids, which was repeated 5 times. The “*” and “**” indicate significant differences (independent *t* test, *p* < 0.05 and *p* < 0.01).

The imidaclothiz has been used extensively to control pests such as aphids, planthoppers, whiteflies, etc. [48]. Based on the plant uptake data, the root application method was firstly used to evaluate the bioactivity of imidaclothiz/SPc complex toward green peach aphids in the laboratory (Figure 4). In the dose-dependent experiments, the mortality of aphids treated with SPc-loaded imidaclothiz was significantly increased by 18.20% (5 mg/mL), 21.37% (3 mg/mL) and 21.77% (1 mg/mL) at 24 h after the treatment, which was consistent with our previous study that the mortality of aphids treated with nanoscale thiamethoxam through the root application was increased by approximately 20% compared with thiamethoxam alone [17]. As expected, the SPc exhibited no obvious toxicity toward aphids, confirming its negligible stomach toxicity. Extremely high concentrations of SPc can down-regulate many membrane protein genes and lysosome genes, leading to the damage of cell membrane in gut tissues of ladybirds [49]. Based on our current data, the SPc exhibited an excellent biocompatibility.

**Figure 5:**
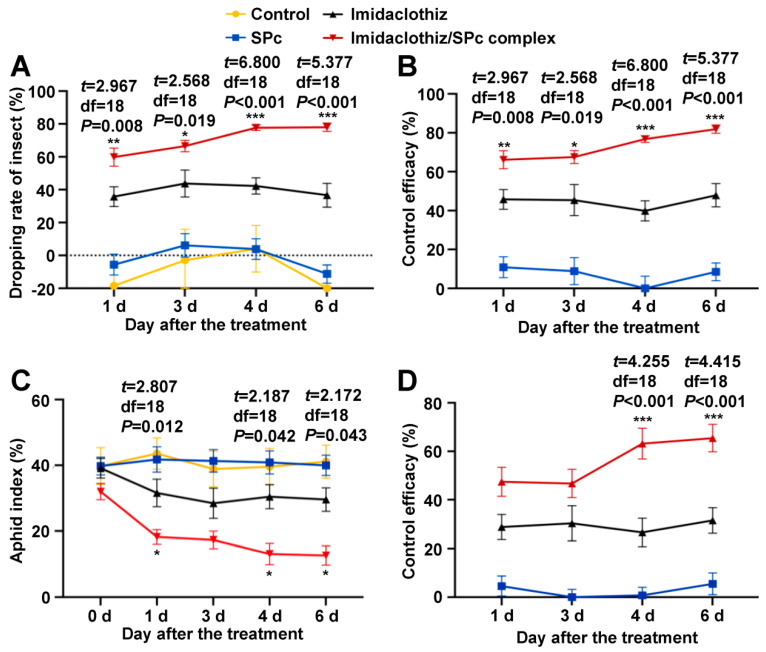
Control efficacy of imidaclothiz/SPc complex toward green peach aphids through spraying method in field. Commercial preparation (cp) of imidaclothiz was mixed with SPc at the mass ratio of 1:1. The 20 and 40 mg/L formulation was sprayed on 0 and 3 d, respectively, with the application amount of 100 mL/m^2^. Sixteen plants from each plot were selected as 16 replicates to record the number of aphids on the top five leaves. Dropping rate of insect (**A**) and control efficacy (**B**) were calculated using method 1, and aphid index (**C**) and control efficacy (**D**) were calculated using method 2. The “*”, “**” and “***” indicate significant differences (independent *t* test, *p* < 0.05, *p* < 0.01 and *p* < 0.001).

Various formulations were sprayed against green peach aphids on tobacco plants in field, and two methods were used to analyze the control efficacy of SPc-loaded imidaclothiz. The dead aphids caused by various formulations exhibited dehydration and turned to black on tobacco leaves (Figure S3). According to the previous studies [10,13], the dropping rate of aphids treated with imidaclothiz/SPc complex was significantly higher than that of imidaclothiz, and the control efficacy of imidaclothiz/SPc complex could reach 76.75% (4 d) and 81.91% (6 d) compared with 39.84% (4 d) and 47.90% (6 d) in imidaclothiz treatment (Figure 5A,B). National standard (grade and investigation method of tobacco diseases and insect pests, GB/T 23222-2008) was also used to analyze the control efficacy. This method also supported the above conclusion that the aphid index in imidaclothiz/SPc complex treatment was consistently lower than that of imidaclothiz treatment, and the control efficacy of SPc-loaded imidaclothiz was significantly increased by 31.69% (4 d) and 28.89% (6 d) (Figure 5C,D). The potential mechanism explaining the enhanced bioactivity may be due to the efficient pesticide nano-delivery system that increases the contact area and plant uptake of pesticides. Similar to a previous study, Zhang et al. [50] constructed the emamectin benzoate (EB) nanogel suspension with a polymer poly (vinyl alcohol)-valine that exhibited higher anti-pest activity than EB emulsifiable concentrate against *Plutella xylostella*, which might be related with the enhanced drug transport across the physiological barriers.

### 2.5. Relative Safety of SPc-Loaded Imidaclothiz

**Figure 6:**
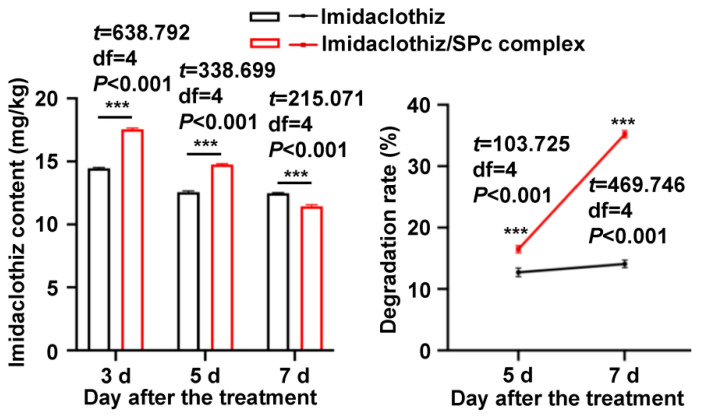
Residue and degradation rate of SPc-loaded imidaclothiz in tobacco plants. Five-leaf stage tobacco plants were immersed in imidaclothiz or imidaclothiz/SPc complex solution for 1 min, and collected on 3, 5 and 7 d after the immersion for liquid chromatography-tandem mass spectrometry (LC-MS/MS) analysis. Each treatment contained 3 independent samples. The “***” indicates significant differences according to the independent *t* test (*p* < 0.001).

The widespread application of neonicotinoids has led to ubiquitous environmental detection, and previous studies have proven the presence of neonicotinoids in various types of bodies of water and soils [51,52,53]. For instance, imidaclothiz is fairly stable in water and soil under natural conditions, and only 25.1% of imidaclothiz can be degraded over a long period of 25 days in soils [54,55]. Whether enhanced plant uptake of SPc-loaded imidaclothiz leads to the higher residue is an inevitable problem before large-scale field application. As shown in Figure 6 and Figure S4, the imidaclothiz residue 3–5 d after the treatment of imidaclothiz/SPc complex was significantly higher than that of imidaclothiz, but the residue was lower on 7 d with the help of SPc. The degradation rate of imidaclothiz alone was 12.67% and 16.15% on 5 and 7 d, and the SPc-loaded imidaclothiz degraded faster with a degradation rate of 13.77% and 34.89%. These results suggested that the SPc could accelerate the degradation of imidaclothiz, which might be due to the faster biodegradation of nanoscale complex in tobacco plants [16,18]. Therefore, the SPc can be applied as a pesticide adjuvant to decrease the pesticide residue and mitigate the negative impacts on the environment. Meanwhile, the status of tobacco plants was observed since the immersion, and no obvious negative effects of SPc-loaded imidaclothiz on plant growth were observed (Figure S5).

**Figure 7:**
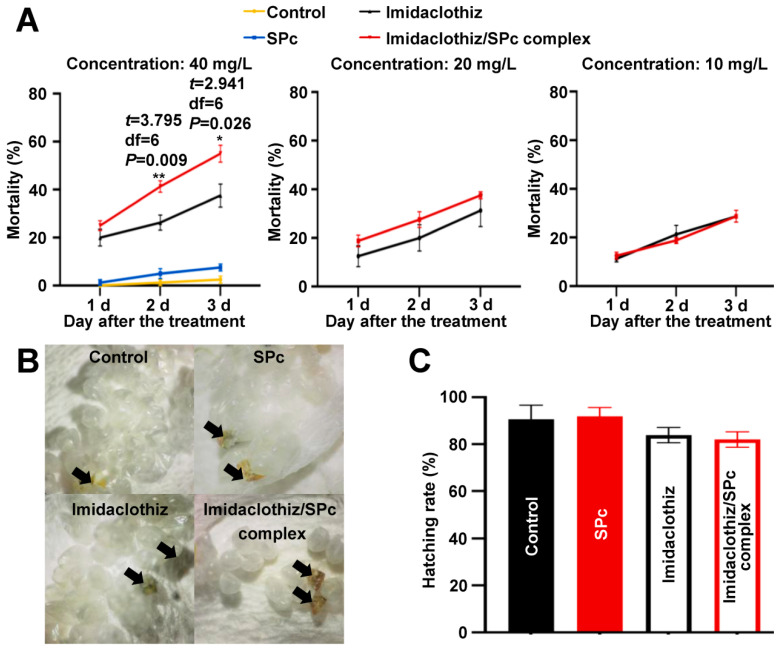
Potential negative effects of SPc-loaded imidaclothiz on lady beetle *Harmonia axyridis*. (**A**) Toxicity of SPc-loaded imidaclothiz against 1st instar larvae of lady beetles. The larvae were immersed in the formulations of imidaclothiz/SPc complex, imidaclothiz, SPc and ddH_2_O (control) for 10 s, and the mortality was recorded and calculated. Each treatment included approximately 20 larvae and was repeated 4 times. The “*” and “**” indicate significant differences according to the independent *t* test (*p* < 0.05 and *p* < 0.01). (**B**) Photos of lady beetle eggs treated with various formulations. The eggs of lady beetles were immersed in the formulations of imidaclothiz/SPc complex at the mass ratio of 1:1 (imidaclothiz concentration: 40 mg/mL), imidaclothiz, SPc and ddH_2_O (control) for 10 s, and the photos were collected 3 d after the immersion. The arrows indicate the dead eggs. (**C**) Egg hatching rate of lady beetles. The hatching rate of above treated eggs was recorded and calculated 3 d after the immersion. Each treatment included approximately 30 eggs and was repeated 4 times.

There is no or very little information about the negative effects of imidaclothiz on non-target organisms. There is only one reference reporting the negative effects of imidaclothiz on earthworms, and the imidaclothiz can induce oxidative damage which causes damage to vital macromolecules [56]. In structure, imidaclothiz has the same imidazolidine ring and nitroguanidine moiety as imidacloprid that exhibits the ecological hazards to earthworms at different organization levels [26,57]. Predatory lady beetles are famous biological agents, and their eggs have been widely released in greenhouses for pest management. The neonicotinoids can influence the performance of lady beetles, which are moderately harmful to the predatory lady beetles [28]. As shown in Figure 7A, the toxicity of SPc-loaded imidaclothiz was slightly improved against the larvae of lady beetles due to the enhancement of broad-spectrum bioactivity. However, the application of imidaclothiz/SPc complex or imidaclothiz exhibited nearly no negative effects on the hatching rate of lady beetles (Figure 7B,C).

## 3. Experimental Methods

### 3.1. Materials

Pure imidaclothiz (≥98%) and cp imidaclothiz (effective content: 10%) were purchased from Macklin Inc. (Shanghai, China) and Nantong Jiangshan Agrochemical Co., Ltd. (Nantong, China), respectively. The 2-bromo-2-methylpropionyl bromide and triethylamine were purchased from Heowns BioChem Technologies (Tianjin, China), the N,N,N′,N′,N″-Pentamethyl diethylenetriamine (PMDETA, 98%) and CuBr (99.999%) were purchased from Sigma-Aldrich (Saint Louis, MO, USA), and the 2-(Dimethyl amino) ethyl methacrylate (DMAEMA, 99%) purchased from Energy Chemical (Shanghai, China) was used to synthesize the star polymer (SPc). Other agents were purchased from Beijing Chemical Works (Beijing, China).

### 3.2. SPc Synthesis

**Figure 8:**
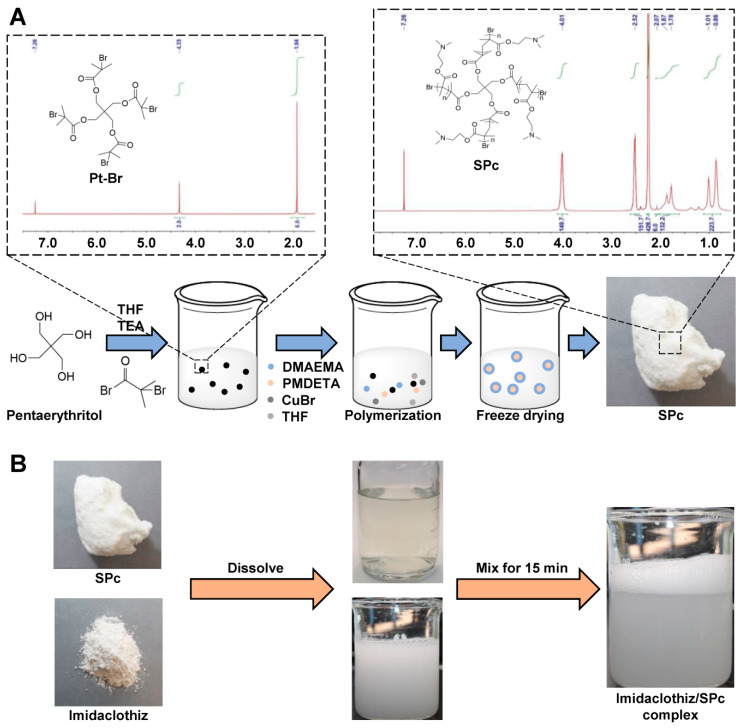
Synthesis route of SPc (**A**) and preparation of imidaclothiz/SPc complex (**B**).

As shown in Figure 8A, the SPc was synthesized according to the method described by Li et al. [9]. In brief, the SPc was synthesized using the commercial and cheap material sources through two reaction steps. The 2-bromo-2-methylpropionyl bromide (253 mg, 1.11 mmol) was added dropwise into the pentaerythritol solution (25 mg, 0.18 mmol) in dry tetrahydrofuran (THF, 20 mL) and triethylamine (TEA, 111.3 mg, 1.11 mmol) at 0°C. The reaction was quenched with methanol after stirring for 24 h at room temperature, and the product was recrystallized in cold ether to obtain the star initiator Pt-Br (50 mg, 40%) that was confirmed by ^1^H NMR (CDCl_3_, Bruker 400, Billerica, Massachusetts, USA). The Pt-Br (40 mg, 0.055 mmol), DMAEMA (2.2 g, 7.7 mmol) and dry THF (8 mL) were added into a flask, and the mixture was degassed by nitrogen for 30 min. The CuBr (46 mg, 0.22 mmol) and PMDETA (110 mg, 0.44 mmol) were then added, and the polymerization was carried out at 60 °C for 7 h. The reaction was quenched by cooling and air exposure, and the THF was removed and recycled for the next polymerization to decrease the production cost. The crude polymer was purified by dialysis in water four times, and the white powder of SPc was finally obtained, which was also confirmed by ^1^H NMR (CDCl_3_, Bruker 400, Billerica, MA, USA).

### 3.3. Construction of Imidaclothiz Nano-Delivery System

As shown in Figure 8B, pure imidaclothiz and SPc were dissolved in double distilled water (ddH_2_O) to prepare the 2 mg/mL of imidaclothiz and SPc aqueous solution, respectively. The imidaclothiz solution was mixed with SPc solution at different mass ratios, and the mixture was incubated for 15 min at room temperature to prepare the imidaclothiz nano-delivery system. The SPc could spontaneously combine with pesticide into pesticide/SPc complex [16,17].

### 3.4. Loading Capacity Measurement

Pure imidaclothiz was dissolved in ddH_2_O to prepare a series of imidaclothiz dilutions (0, 8, 11, 14, 17 and 20 μg/mL), and the ultraviolet absorbance was determined via UV-vis spectrophotometry (Thermo Genesys180, Saint Louis, MO, USA). The standard calibration curve was constructed using the absorbance at 270 nm. The 2 mL of excess imidaclothiz solution (0.25 mg/mL) was mixed with 2 mL of SPc solution (0.304 mg/mL) to determine the pesticide loading content (PLC). The mixture incubated for 15 min was dialyzed using the regenerated cellulose with a molecular weight cutoff of 1000 Da (Shanghai Yuanye Bio-Technology Co., Ltd., Shanghai China) for 12 h. The absorbance at 270 nm was measured to determine the imidaclothiz concentration, and the PLC was calculated using the formula of PLC (%) = weight of imidaclothiz loaded in complex ÷ weight of imidaclothiz-loaded complex × 100%.

### 3.5. Isothermal Titration Calorimetry (ITC) Assay

As a universal method, ITC is a high-accuracy method for measuring binding affinities [58,59], which was performed to examine the binding force between imidaclothiz and SPc. The 2 mL of pure imidaclothiz solution (0.138 mmol/L) was titrated with 250 μL of SPc solution (1 mmol/L) in Nano ITC (TA Instruments Waters, New Castle, DE, USA). The heats of interaction during each injection were calculated by integrating each titration peak using Origin7 software (OriginLab Co., Ltd., Northampton, MA, USA). The test temperature was 25 °C, and ΔG was calculated using the formula of ΔG = ΔH − TΔS.

### 3.6. Particle Size Measurement and Complex Morphology Characterization

Pure imidaclothiz was mixed with SPc at the mass ratios of 1:1, 1:2 and 1:3 to prepare the imidaclothiz/SPc complex, respectively. The particle sizes of imidaclothiz and imidaclothiz/SPc complex at various mass ratios were measured using a Particle Sizer and Zeta Potential Analyzer (Brookhaven NanoBrook Omni, New York, NY, USA) at 25 °C. Each treatment contained 3 independent samples. The morphologies of imidaclothiz and imidaclothiz/SPc complex at the mass ratio of 1:1 were further examined using a transmission electron microscope (TEM, JEOL-1200, Tokyo, Japan). A 10 μL of each sample was dropped on the microgrid and treated with 2% phosphotungstic acid. Two samples were air-dried before the observation.

### 3.7. Contact Angle Analysis

The contact angles of pure imidaclothiz and imidaclothiz/SPc complex at the mass ratio of 1:1 were examined to evaluate the wetting performance using an Optical Contact Angle Meter (Date Physics Corporation OCA25, Stuttgart, Germany) according to the method described by Zhu et al. [60]. The SPc and ddH_2_O were applied as controls. The 5 μL of various samples (1 mg/mL) was dripped onto the glass slide, and the image of contact angle between the liquid and glass slide was collected when the droplet became stable for approximately 10 s. The contact angle was analyzed using the ellipse fitting algorithm [61]. The algorithm assumes that the water drop profile is part of an ellipse. Each treatment included 3 independent samples.

### 3.8. Plant Uptake Analysis

Cp imidaclothiz was mixed with SPc at the mass ratio of 1:1 to prepare imidaclothiz/SPc complex solution (imidaclothiz concentration: 40 mg/L), which was used to test the plant uptake. Five-leaf stage tobacco plants (*Nicotiana benthamiana*) were immersed in imidaclothiz or imidaclothiz/SPc complex solution for 1 min, and then air-dried. The plant uptake needed to be examined in a relatively short time; thus the plants were washed with ddH_2_O to remove the pesticide on the plant surface at 1, 6 and 12 h after the immersion, and collected for liquid chromatography-tandem mass spectrometry (LC-MS/MS) analysis. The ddH_2_O was used as control. Each treatment included 3 independent samples.

The extraction and quantification of imidaclothiz were similar to the procedure described by Jiang et al. [18]. In brief, the imidaclothiz was extracted from homogenized plants (5 g) using 20 mL acetonitrile acetate (1%). After the centrifugation, the 20 mL of supernatant was evaporated using nitrogen (40 °C) until the volume was reduced to 1 mL, which was purified using a polytetrafluoroethylene membrane filter (Haiming Zhongli Filtering Equipment Factory, Haining, China). The obtained residues were dissolved in 1 mL acetonitrile/water (2:8 *v*/*v*) for LC-MS/MS analysis, which was performed on an ACQUITY UPLC-TQD system (Waters Co., Milford, Massachusetts, USA) with a Shim-Pack GIST C18 column (2 μm, 2.1 × 100 mm, Shanghai, China). The two analytes were separated using a mobile phase consisting of acetonitrile-0.1% formic acid (2:8 *v*/*v*) solution. The injection volume was 20 μL, and the column temperature was 40 °C.

### 3.9. Bioactivity Evaluation through Root Application in Laboratory

According to the PLC, pure imidaclothiz was mixed with SPc at the mass ratio of 1:5.1 to prepare the imidaclothiz/SPc complex (imidaclothiz concentration: 5, 3 and 1 mg/mL). The root application was applied to examine the bioactivity of imidaclothiz/SPc complex toward green peach aphids that pierce the phloem and indirectly transmit plant virus in many crops [62]. Similar to the methods described by Deng et al. [63] and Zhang et al. [64], the roots of 9–10 cm height radish seedlings infested with aphids (about 30 aphids per plant) were immersed in the formulations of imidaclothiz and imidaclothiz/SPc complex. The highest concentrations of SPc and ddH_2_O were employed as controls. The treated aphids were maintained at 18 ± 1 °C, 80 ± 10% relative humidity and 14L: 10D photoperiod in an incubator. The number of dead aphids was recorded at 12, 24 and 36 h after the treatment, and mortality was calculated. Each treatment was repeated 5 times.

### 3.10. Bioactivity Evaluation through Spraying Application in Field

Cp imidaclothiz was mixed with SPc at the mass ratio of 1:1 to prepare the imidaclothiz/SPc complex (imidaclothiz concentration: 20 and 40 mg/L). The spraying experiment was carried out against green peach aphids in tobacco field. The 20 mg/L of imidaclothiz and imidaclothiz/SPc complex was firstly sprayed on 0 d using an electric 528B (Shenzhen Longray Tech. Co., Shenzhen, China) with the application amount of 100 mL/m^2^, and the 40 mg/L of imidaclothiz and imidaclothiz/SPc complex was sprayed on 3 d again. The 200 and 400 mg/L of SPc were also sprayed on 0 and 3 d, respectively. The ddH_2_O was applied as control. The area of each plot was 40 m^2^ and each plot contained approximately 80 plants. Sixteen plants from each plot were selected as 16 replicates to record the number of aphids on the top five leaves on 0, 1, 3, 4 and 6 d.

Two methods were used to calculate the control efficacy. (1) According to the previous studies [10,13], the dropping rate of insect (DRI) and control efficacy (CE) were calculated using the formulas of DRI (%) = (aphid number before pesticide application − aphid number after pesticide application) ÷ aphid number before pesticide application × 100% and CE (%) = (DRI in the treatment plot − DRI in the control plot) ÷ (100 − DRI in the control plot) × 100%. (2) According to the national standard (grade and investigation method of tobacco diseases and insect pests, GB/T 23222-2008), the plants infested with aphids were classified. Grade 0: no aphid; Grade 1: 1~5 aphids/leaf; Grade 3: 6~20 aphids/leaf; Grade 5: 21~100 aphids/leaf; Grade 7: 101~500 aphids/leaf; Grade 9: >500 aphids/leaf. The aphid index (AI) and control efficacy (CE) were calculated using the formulas of AI (%) = Σ (number of leaves infested with aphids × grade) ÷ (number of investigated leaves × 9) × 100% and CE (%) = (1 − AI in treatment plot after pesticide application × AI in control plot before ddH_2_O application) ÷ (AI in treatment plot before pest application × AI in control plot after ddH_2_O application) × 100%.

### 3.11. Safety Assessment of SPc-Loaded Imidaclothiz

Neonicotinoid residue has been a major public concern around the world and is directly related to food and environmental safety [27,64]. The degradation rate of imidaclothiz is relatively low compared to that of acetamiprid or thiacloprid, which may pose a potential risk to human health [65]. To examine the residue of SPc-delivered imidaclothiz, cp imidaclothiz was mixed with SPc at the mass ratio of 1:1 to prepare imidaclothiz/SPc complex (imidaclothiz concentration: 40 mg/L). Five-leaf stage tobacco plants were immersed in imidaclothiz or imidaclothiz/SPc complex solution for 1 min, and collected on 3, 5 and 7 d after the immersion for LC-MS/MS analysis. Each treatment included 3 independent samples. The degradation rate (DR) was calculated using the formula of DR (%) = (imidaclothiz content on 3 d − imidaclothiz content on 5/7 d) ÷ imidaclothiz content on 3 d × 100%. Meanwhile, considering the potential chemical damage brought by SPc-delivered pesticides, the status of the above treated plants was observed since the immersion, and the plant height, plant weight, and the largest leaf length and width were measured on 0, 3, 5 and 7 d after the immersion. Six plants were selected as 6 replicates to record the data.

As a major predator of aphids, lady beetle *H. axyridis* was selected to evaluate the toxicity of SPc-loaded imidaclothiz. The eggs of lady beetles were immersed in pure imidaclothiz/SPc complex at the mass ratio of 1:1 (imidaclothiz concentration: 40 mg/mL), pure imidaclothiz, SPc and ddH_2_O (control) for 10 s, and the hatching rate was recorded and calculated on 3 d after the immersion. Meanwhile, the first instar larvae were treated similar as above, and the mortality was recorded and calculated on 1, 2 and 3 d after the immersion. Each treatment included approximately 30 eggs or 20 larvae, and was repeated 4 times.

### 3.12. Data Analysis

The Tukey HSD test or independent *t* test was conducted using SPSS 26.0 (SPSS Inc., New York, NY, USA) at the *p* = 0.05 level of significance. The descriptive statistics were shown as the mean value and standard errors of the mean.

## 4. Conclusions

Herein, a relatively safe imidaclothiz nano-delivery system was constructed successfully based on a star polymer. The imidaclothiz could be loaded in the hydrophobic core of SPc spontaneously through hydrophobic association. This self-assembly formed nearly spherical particles of imidaclothiz/SPc complex with nanoscale size. The contact angle of imidaclothiz decreased with the help of SPc, suggesting the easier distribution and spreading of imidaclothiz/SPc complex. Furthermore, the plant uptake of SPc-loaded imidaclothiz was remarkably increased and thus its bioactivity and control efficacy were significantly improved against green peach aphids in both laboratory and field. Excitingly, the SPc-loaded imidaclothiz degraded faster than imidaclothiz alone in tobacco plants due to the smaller particle size. In addition, the imidaclothiz/SPc complex exhibited no negative effects on the agronomic traits of tobacco plants but had a slight synergistic effect on predatory lady beetles. The current study has constructed a pesticide nano-delivery system for improved plant uptake, reduced residue and enhanced bioactivity, which is beneficial for pesticide reduction in sustainable agriculture.

## Figures and Tables

**Table 1:** Reduced particle size of SPc-loaded imidaclothiz at various mass ratios.

Formulation	Mass Ratio	Sample Number	Size (nm)	Average Size (nm)
Imidaclothiz	-	1	182.00	187.76 ± 6.31 a
		2	186.76	
		3	194.51	
Imidaclothiz/SPc complex	1:1	1	82.12	84.28 ± 2.04 b
		2	84.55	
		3	86.18	
	1:2	1	64.55	60.76 ± 3.28 c
		2	58.94	
		3	58.80	
	1:3	1	43.67	44.84 ± 1.26 d
		2	44.67	
		3	46.18	
*F_3,8_* = 879.818, *p* < 0.001				

Means ± SE followed by different letters are significantly different (Tukey HSD test, *p* < 0.05).

## Data Availability

All data in this study will be available from the corresponding author upon reasonable request.

## Supplementary Materials

The following supporting information can be downloaded at: https://www.mdpi.com/article/10.3390/ijms23126651/s1.
