# Supplementary material for: Nanocarrier-Loaded Imidaclothiz Promotes Plant Uptake and Decreases Pesticide Residue
Source: Int J Mol Sci. 2022 Jun 14;23(12):6651. doi: 10.3390/ijms23126651 (PMC9224201; doi:10.3390/ijms23126651)
Supplement: Supplementary file 1 [file ijms-23-06651-s001.zip › ijms-1739026-supplementary.pdf]

Supporting Information

Nanocarrier-Loaded Imidaclothiz Promotes Plant Uptake and Decreases Pesticide Residue

Qinhong Jiang [1], Min Peng [2], Meizhen Yin [2], Jie Shen [1,*] and Shuo Yan [1,*]

[1] Department of Plant Biosecurity and MARA Key Laboratory of Surveillance and Management for Plant Quarantine Pests, College of Plant Protection, China Agricultural University, Beijing, China; J.yucheng@outlook.com (Q.J.)

[2] State Key Laboratory of Chemical Resource Engineering, Beijing Lab of Biomedical Materials, Beijing University of Chemical Technology, Beijing, China; 2020400119@buct.edu.cn (M.P.); yinmz@mail.buct.edu.cn (M.Y.)

* Correspondence: shenjie@cau.edu.cn (J.S.); yanshuo2011@foxmail.com (S.Y.)

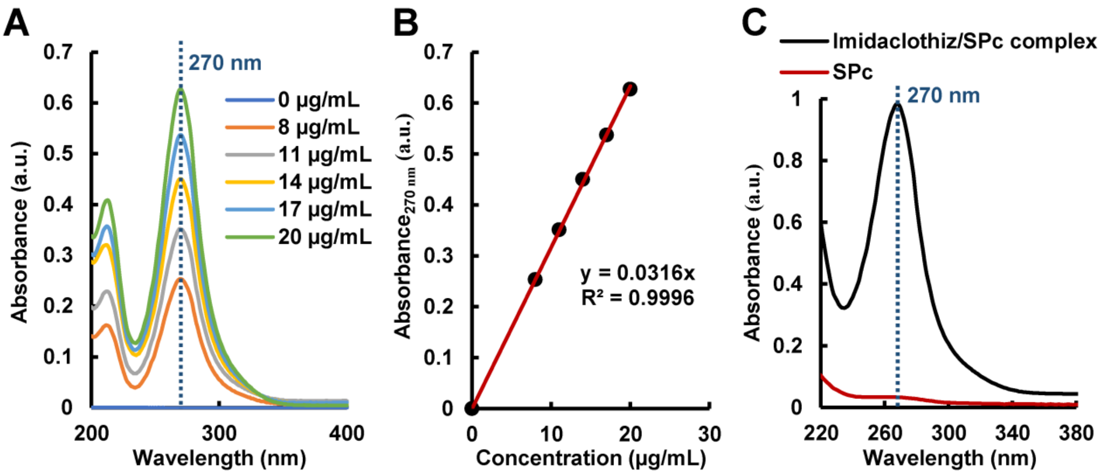

Figure S1. Loading capacity of SPc toward imidaclothiz. (A) Absorbance spectra of imidaclothiz with various concentrations. (B) Standard calibration curve of imidaclothiz. (C) Absorbance spectra of imidaclothiz/SPc complex and SPc.

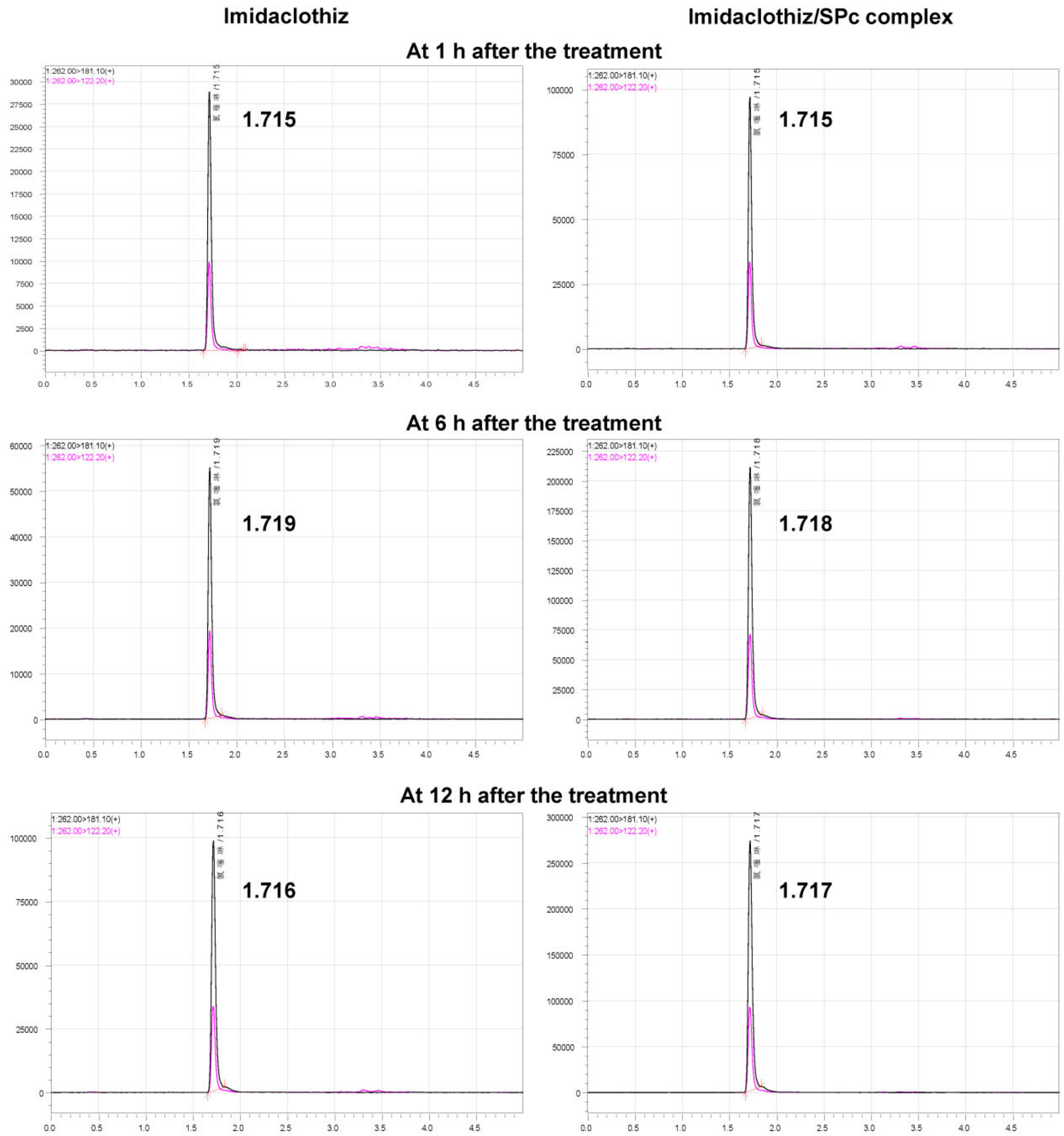

Figure S2. Original spectra of liquid chromatography-tandem mass spectrometry data for determining the plant uptake of imidaclothiz by tobacco plants.

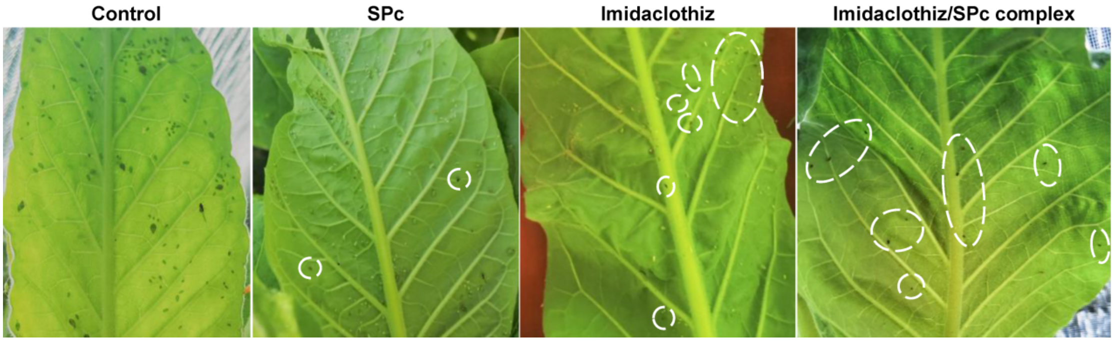

Figure S3. Photos of lethal phenotype in various treatments. White circles indicate the dead aphids.

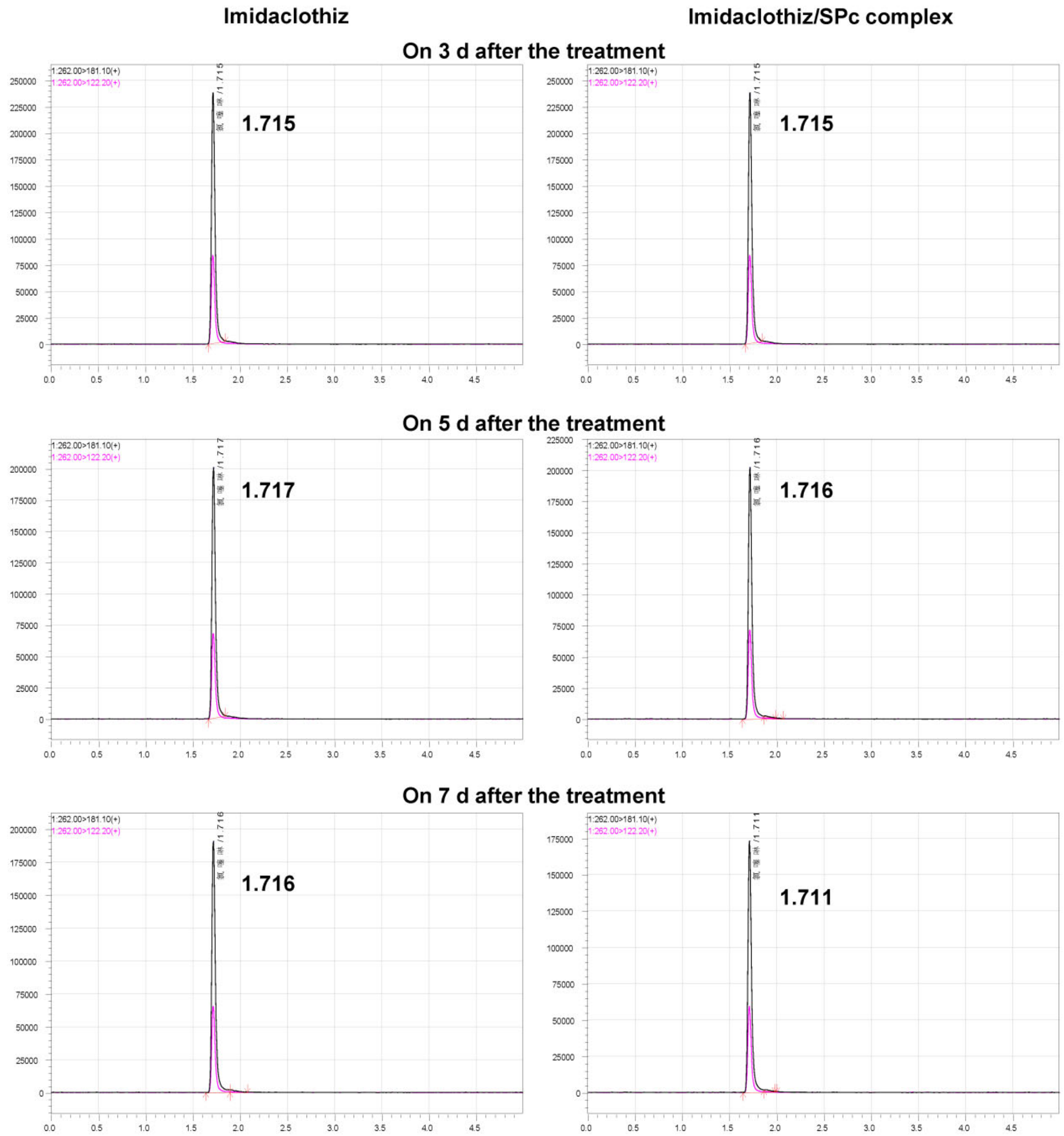

Figure S4. Original spectra of liquid chromatography-tandem mass spectrometry data for determining the imidaclothiz residue in tobacco plants.

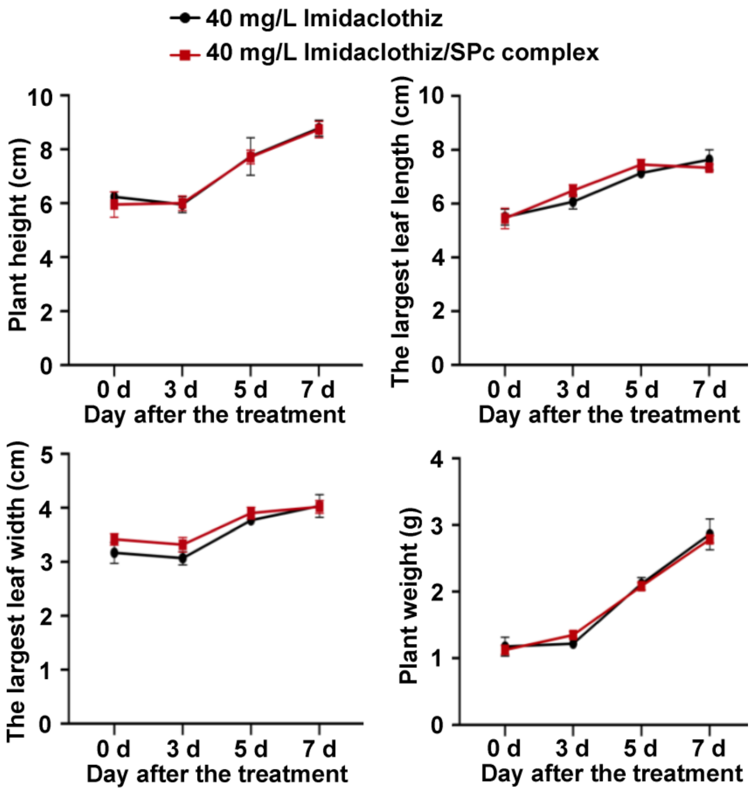

Figure S5. Effects of imidaclothiz/SPc complex on the agronomic traits of tobacco plants. Six plants were selected as 6 replicates to record the data.
